# Role of anti-Müllerian hormone and testosterone in follicular growth: a cross-sectional study

**DOI:** 10.1186/s12902-020-00569-6

**Published:** 2020-07-08

**Authors:** Ping-Ping Lv, Min Jin, Jin-Peng Rao, Jian Chen, Li-Quan Wang, Chang-Chang Huang, Song-Qing Yang, Qiu-Ping Yao, Lei Feng, Jin-Ming Shen, Chun Feng

**Affiliations:** 1 The Women’s Hospital of Zhejiang University School of Medicine, Hangzhou, 310006 Zhejiang China; 2 The Second Affiliated Hospital of Zhejiang University School of Medicine, 88 Jiefang Road, Hangzhou, 310009 Zhejiang China; 3 The First Affiliated Hospital of Zhejiang Chinese Medicine University, Hangzhou, 310006 Zhejiang China

**Keywords:** Anti-Müllerian hormone (AMH), Diminished ovarian reserve (DOR), Polycystic ovarian syndrome (PCOS), Follicle-stimulating hormone (FSH), Luteinizing hormone (LH), Testosterone

## Abstract

**Background:**

Anti-Müllerian hormone (AMH) is now considered the best serum biomarker of ovarian reserve, while basal sex hormones are classic markers used for assessing ovarian reserve. The interaction between AMH and sex hormones are complicated and not sufficiently addressed. In this study, we took diminished ovarian reserve (DOR) and polycystic ovarian syndrome (PCOS) as two extremes of ovarian reserve (deficient and excessive respectively) to investigate the role of AMH and sex hormones in follicular growth.

**Methods:**

A retrospective cross-sectional survey was performed. The patients assessed AMH and basal sex hormones in the Second Hospital of Zhejiang University from April 2016 to March 2019 were involved in this study. Serum AMH and sex hormone concentrations were tested with electrochemiluminescence method. Stepwise linear regression and binary logistic regression was used to determine the predictors of AMH level and to explore the involved factors determining DOR and PCOS.

**Results:**

In the present study, we found that age and follicle-stimulating hormone (FSH) were main negative correlation factors, and luteinizing hormone (LH) and testosterone (T) were main positive factors of AMH. In DOR group, age, FSH and estradiol (E_2_) increased and T decreased, while in PCOS group, LH and T increased. Binary logistic regression found that age, weight, FSH, E_2_, and T were the significant factors which independently predicted the likelihood of DOR, and that age, body mass index (BMI), AMH, LH, and T predicted the likelihood of PCOS.

**Conclusions:**

Our study demonstrated that age, FSH, and T were factors that most closely correlated with AMH level, and T was involved in both DOR and PCOS. Since DOR and PCOS are manifested with insufficient AMH and excessive AMH respectively, it is suggested that total testosterone correlated with AMH closely and plays an important role in follicular growth. More attention should be given to testosterone level during controlled ovarian hyperstimulation (COH) process.

## Background

Gonadal hormone anti-Müllerian hormone (AMH), a member of transforming growth factor-β (TGF-β) superfamily, is a glycoprotein synthesized by granulosa cells [1]. It is now considered the best serum biomarker of ovarian reserve, reflecting the number of primordial follicles and its response to exogenous gonadotropins [2]. It is generally agreed that intra-individual variation of serum AMH is less than true biological variability [3, 4], and AMH level is not disturbed by menstrual cycles and exogenous sex steroids [5], so the test of AMH can be performed on any day of menstrual cycle, which is much more convenient than basal follicle-stimulating hormone (FSH) test. Nowadays AMH is widely used in menopause prediction, predicting pregnancy chances in infertility couples, management in IVF, and management of women with cancer [1].

Some studies investigated the relationship between AMH and gonadotropins. It was found that after gonadotropin-releasing hormone agonist (GnRHa) treatment AMH levels decreased significantly together with increase of FSH and luteinizing hormone (LH), and then all hormone levels reversed, in which there was a moderate negative correlation between AMH and FSH [6]. However, others reported that after GnRHa administration AMH level changed significantly, and changes of AMH levels did not correlate with changes of gonadotropins, estradiol, or progesterone [7]. Evenmore, it was found that AMH receptor type 2 (AMHR2) was expressed in pituitary gonadotrope cells and AMH directly stimulated the secretion of FSH and LH [8–10]. In all, the relationship between AMH and sex hormones is complicated and inconsistent, further investigation is required to address the correlation.

AMH inhibits follicle development in two critical points: the recruitment of primordial follicles into the pool of growing follicles and the responsiveness of growing follicles to FSH [11–13]. An assessment of the correlation of AMH and individual sex hormone would be valuable for understanding the regulatory function of AMH in follicle development. Diminished ovarian reserve (DOR) and polycystic ovarian syndrome (PCOS) are representative of two extremes of ovarian reserve [14, 15], to explore the predictive factors of DOR and PCOS helps to understand the regulation mechanism of follicle development.

The aim of the present study was to determine the association between serum AMH level and serum basal sex hormone concentrations. Firstly, correlation analysis was performed to explore the individual correlation between AMH and basal sex hormones, and further multiple regression analysis was carried out to select predictive determinants of serum AMH level. Secondly, sex hormone levels were compared in DOR and PCOS populations, and logistic regression analysis was used to further determine the predictive factors of DOR and PCOS.

## Methods

### Study population

**Fig. 1:**
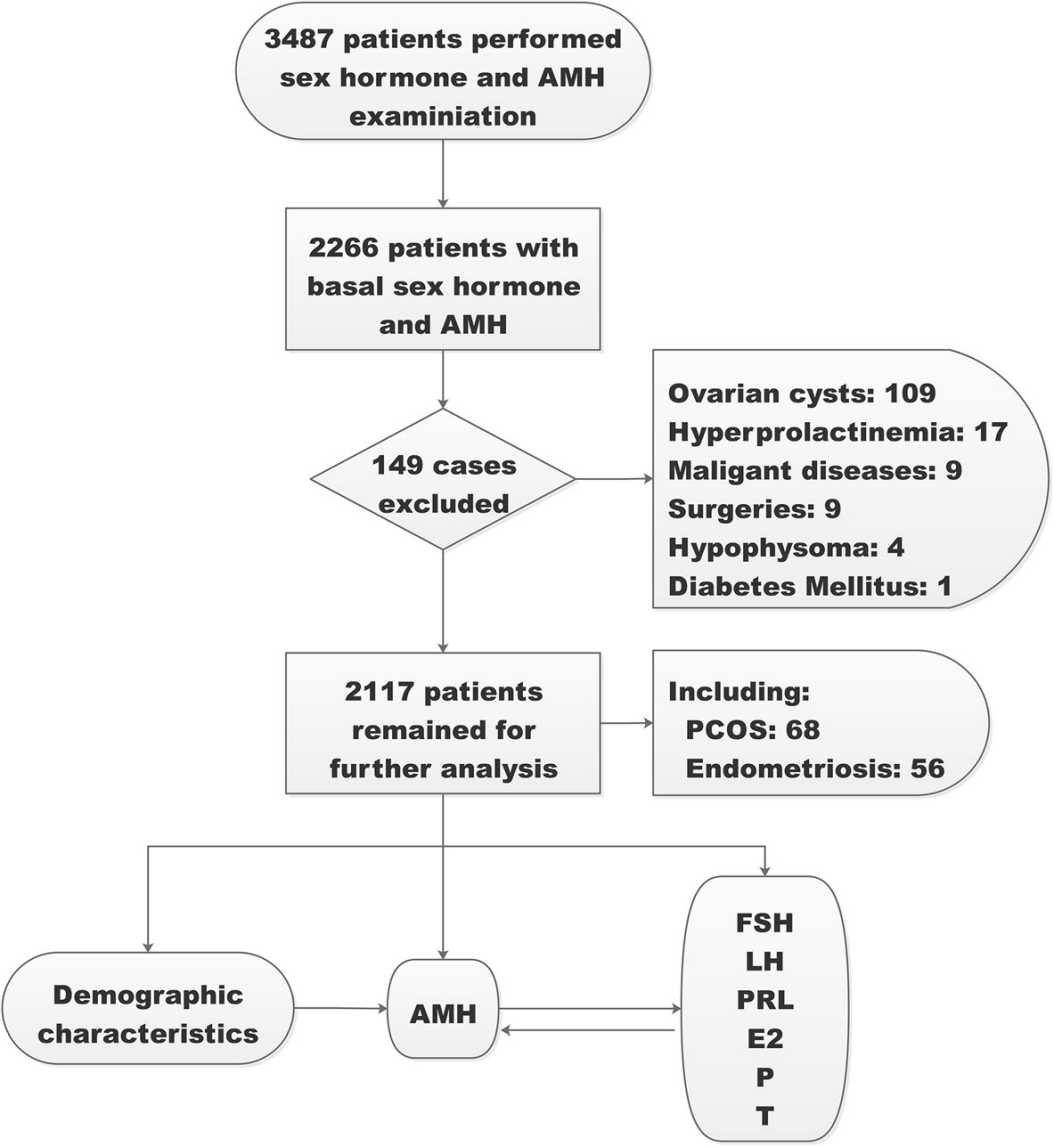
Work flow. Of 3487 women with both AMH and sex hormones assessment, 1221 cases were excluded because the sex hormones assessment was not performed in early follicle phase, and 149 cases were excluded for ovarian cysts, hyperprolactinemia and so on. Finally 2117 women were remained for further analyses. PCOS: polycystic ovarian syndrome. AMH: anti-Müllerian hormone. FSH: follicle-stimulating hormone. LH: luteinizing hormone. PRL: prolactin. E_2_: estradiol. P: progesterone. T: testosterone

This was a retrospective cross-sectional study for correlation between AMH and sex hormones in women attending for pre-pregnancy examination or infertility at the Second Hospital of Zhejiang University School of Medicine. This study adhered to standard biosecurity and institutional safety procedures. As shown in Fig. 1, the patients performed AMH and basal sex hormones examination from April 2016 to March 2019 were included in this study. The specialists of reproductive medicine or gynecology recorded the demographic characteristics, carried out the examinations, and made diagnosis. Exclusion criteria included: 1) ovarian cysts, 2) hyperprolactinemia, 3) malignant diseases, 4) surgeries, 5) hypophysoma, and 6) diabetes mellitus.

### Outcome measurements

Demographic characteristics and diagnosis were recorded in the database system. Serum AMH and sex hormones levels were measured with electrochemiluminescence method by Elecsys® AMH, FSH, LH, prolactin (PRL), estradiol (E_2_), progesterone (P), testosterone (T) from Roche Diagnostics. Fasting plasma glucose (FPG) was determined by commercial enzymatic methods from Shanghai Rongsheng Biotech. Fasting plasma insulin (FPI) was determined with chemiluminescence from SIEMENs Healthcare Diagnostics. AMH is an effective test predicting ovarian reserve, and low AMH cutpoint (0.2–0.7 ng/ml) have been found to have ideal sensitivity and specificity for DOR [16]. In the present study diagnosis of DOR was made when AMH was less than 0.7 ng/ml. According to Chinese Guidelines for Diagnosis and Treatment of Polycystic Ovary Syndrome (Chinese Society of Obstetrics and Gynecology Endocrinology Group and Guidelines Expert Group, 2018) [17], the diagnosis of PCOS was based on the presence of oligomenorrhea/amenorrhea, and at least one of the following criteria: hyperandrogenaemia/hyperandrogenism and polycystic ovaries.

### Statistics

Analyses were performed with the use of SPSS 19.0 statistics package (SPSS, Chicago, IL, USA). Continuous variables were presented as mean values ± SD. One-way ANOVA was used for compares between groups, and Tukey post hoc pairwise comparison was performed with significant difference. Pearson correlation analysis was adopted to analyze the relationship between two variables. Stepwise linear regression was used to determine the significant predictors of serum AMH level. Binary logistic regression with forward method was conducted to explore the involved factors determining DOR and PCOS. A *P* value of < 0.05 was considered as statistically significant.

## Results

### Description of the study sample

As shown in Fig. 1, from April 2016 to March 2019 a total of 3487 women examined with AMH and sex hormones were included. One thousand two hundred twenty-one cases were excluded because the sex hormone test was not taken in early follicle phase. Later 149 cases were excluded for ovarian cysts, hyperprolactinemia, malignant diseases, surgeries, hypophysoma, and diabetes mellitus. Finally 2117 women were remained for further analysis of correlation, including 68 PCOS and 56 endometriosis.

The characteristics of the study population are presented in Table 1. In the DOR group there were higher mean age, weight and BMI (*P* < 0.001). In PCOS group the mean age was significantly lower in PCOS group than in control group, and the mean weight, BMI, and AMH were significantly higher (P < 0.001). There was no difference in height, FPG, and FPI levels (*P* > 0.05).

**Table 1:** Characteristics of the included subjects

Characteristics	All	Control	DOR	PCOS	P	
Cases (N)	2117	1714	335	68		
Age (years)	32.02 ± 6.80	30.51 ± 5.54	40.67 ± 6.55	27.71 ± 3.43	< 0.001	
Weight (kg)	54.34 ± 8.01	53.83 ± 7.99	56.06 ± 7.39	58.83 ± 9.14	< 0.001	
Height (cm)	160.90 ± 4,81	160.94 ± 4.85	160.62 ± 4.65	161.22 ± 4.60	0.455	
BMI (kg/m^2^)	20.98 ± 2,86	20.77 ± 2.84	21.72 ± 2.63	22.60 ± 3.19	< 0.001	
AMH (ng/ml)	3.48 ± 3.09	4.08 ± 3.00	0.29 ± 0.22	6.93 ± 3.84	< 0.001	
FPG (mmol/L)	5.01 ± 0.29	5.02 ± 0.30	4.99 ± 0.28	4.97 ± 0.28	0.288	
FPI (pmol/L)	90.28 ± 66.66	91.31 ± 67.48	75.80 ± 55.39	96.14 ± 54.34	0.565	

Data were presented as mean ± SD

*DOR* Decreased ovarian reserve. *AMH* Anti-Müllerian hormone. *PCOS* Polycystic ovarian syndrome. *BMI* Body mass index. *FPG* Fasting plasma glucose. *FPI* Fasting plasma insulin

### Correlation between demographic characteristics, sex hormones and AMH

**Fig. 2:**
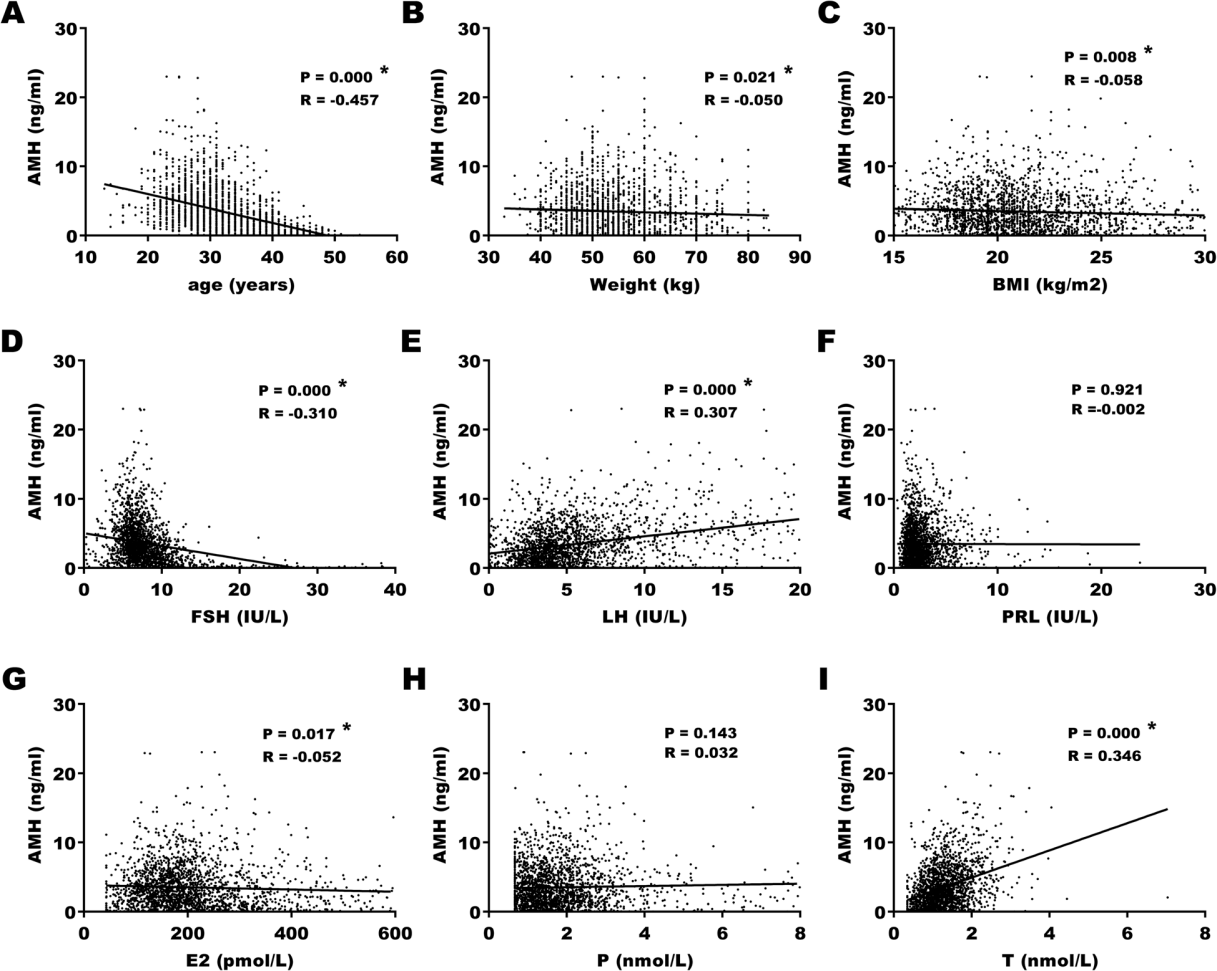
Correlation between AMH level and demographic characteristics and sex hormones. AMH: anti-Müllerian hormone. BMI: body mass index. FSH: follicle-stimulating hormone. LH: luteinizing hormone. PRL: prolactin. E_2_: estradiol. P: progesterone. T: testosterone. * *P* < 0.05

Pearson test was carried out to investigate the correlation between demographic characteristics, gonadotropins, ovarian steroids and AMH. As shown in Fig. 2a and d, in total population, age (R = − 0.457, *P* < 0.001) and FSH (R = − 0.310, *P* < 0.001) were moderately and mildly correlated with AMH negatively. Meanwhile, LH (R = 0.307, P < 0.001) and T (R = 0.346, P < 0.001) were mildly correlated with AMH positively (Fig. 2e and i). There were weak negative correlation between weight (R = − 0.050, *P* = 0.021), BMI (R = − 0.058, *P* = 0.008), E_2_ (R = − 0.052, *P* = 0.017), and AMH (Fig. 2b, c, and g). There was no correlation between PRL, P, height and AMH (*P* > 0.01).

Since Pearson correlation analyses indicated that age, weight, BMI, FSH, LH, E_2_, and T were correlated with AMH, multiple linear regression analyses with stepwise analyses were performed to further address the relationship between these indicators and AMH. The results showed that in the control group, significant contributions for the prediction of serum AMH were provided by age, FSH, LH, E_2_, and T but not weight and BMI (Table 2). Age, FSH, and E_2_ negatively correlated with AMH and while LH and T positively correlated with AMH (*P* < 0.001).

**Table 2:** Correlation of demographic characteristics, sex hormones, and AMH by multiple linear regression

Variables	B	Standard error	LCL	UCL	P value
age	−0.117	0.009	−0.134	−0.100	< 0.001
LH	0.292	0.016	0.260	0.323	< 0.001
FSH	−0.199	0.012	−0.223	− 0.175	< 0.001
E_2_	−0.005	0.001	−0.006	− 0.004	< 0.001
T	0.857	0.106	0.649	1.065	< 0.001

*LCL* 95% lower confidence limit. *UCL* 95% upper confidence limit. *LH* Luteinizing hormone. *FSH* Follicle-stimulating hormone. *E*_*2*_ Estradiol. *T* Testosterone

### Demographic characteristics and sex hormones in DOR group

**Fig. 3:**
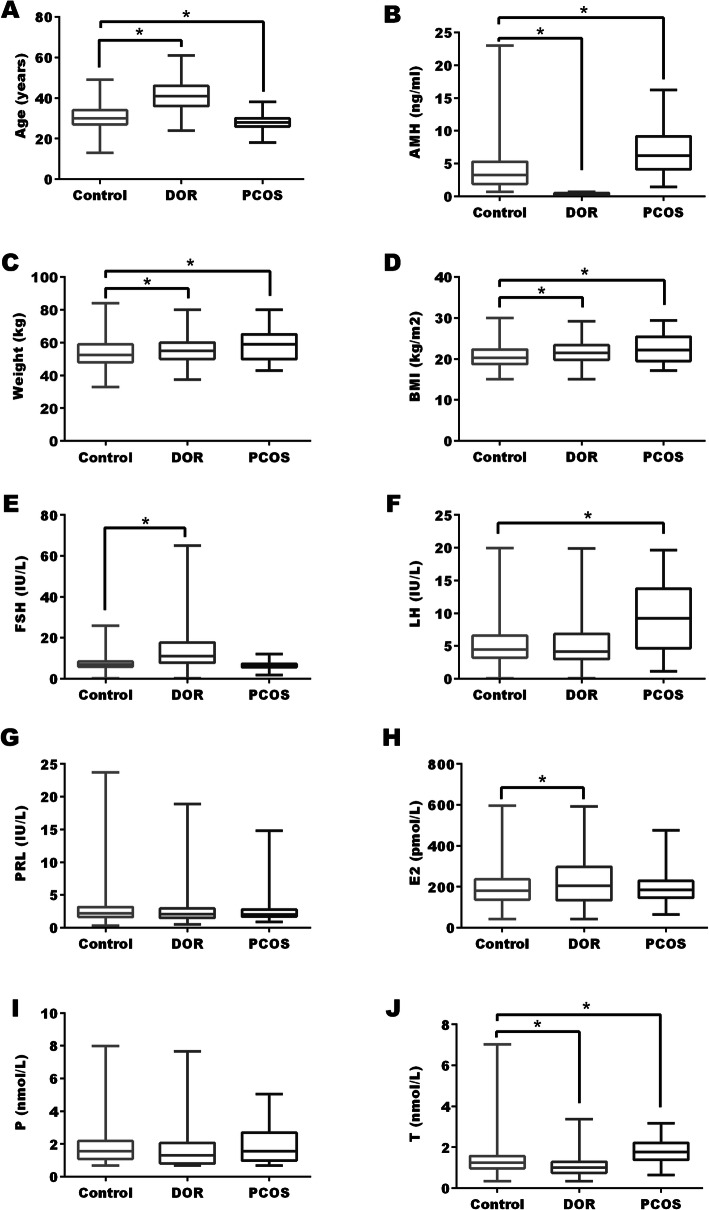
Demographic characteristics and sex hormones in NOR, DOR, and PCOS groups. NOR: normal ovarian reserve. DOR: diminished ovarian reserve. PCOS: polycystic ovarian syndrome. BMI: body mass index. FSH: follicle-stimulating hormone. LH: luteinizing hormone. PRL: prolactin. E_2_: estradiol. P: progesterone. T: testosterone. * *P* < 0.05

Age was significantly older in DOR group in comparison to control (Fig. 3a). Since DOR was diagnosed with AMH cutoff in this study, the AMH level in DOR group was much lower than control group (Fig. 3b). Weight and BMI showed slightly increase in DOR group (*P* < 0.05) (Fig. 3c and d). For pituitary hormones, FSH level increased significantly in DOR group, while LH and PRL did not alter significantly between two groups (*P* > 0.05) (Fig. 3e, f, and g). For ovarian steroids, a significant increase of E_2_ and a significant decrease of T was observed in DOR groups when compared to control (Fig. 3h, h, and j).

### Demographic characteristics and sex hormones in PCOS group

Age was significantly lower in PCOS group compared with control (Fig. 3a). As shown in Fig. 3b, mean AMH level nearly doubled in PCOS group compared to control group (6.93 ± 3.84 vs. 4.08 ± 3.00, *P* < 0.05). Weight and BMI levels revealed a significant increase in PCOS group when compared with control (Fig. 3c and d). For pituitary hormones, LH level increased significantly in PCOS group, and FSH and PRL levels were similar between the two groups (Fig. 3e, f and g). For ovarian hormones, T level showed a significant rise in PCOS group when compared to control, and variation in E_2_ and P were not significant (Fig. 3h, i, and j).

### Logistic regression of DOR and PCOS groups

Binary logistic regression with forward method was used to analyze the prediction on DOR by age, weight, BMI, FSH, E_2_ and T. Age, weight, FSH, E_2_, and T, but not BMI, were the significant factors which independently predicted the likelihood of DOR. In the logistic regression model adjusted for age, weight, FSH and E_2_, one nmol/L increase of T level was associated with 0.60 times decreased (95% CI: 0.42–0.87, *P* = 0.007) risk of DOR (Table 3).

**Table 3:** Relationship of AMH, demographic characteristics, and sex hormones in DOR and PCOS patients using logistic regression

DOR				PCOS			
Variables	OR	95% CI	*P* value	Variables	OR	95% CI	*P* value
age	1.25	1.22–1.29	< 0.001	age	0.92	0.88–0.97	0.002
weight	1.02	1.00–1.05	0.041	BMI	1.23	1.14–1.34	< 0.001
FSH	1.32	1.25–1.38	< 0.001	AMH	1.12	1.05–1.19	0.001
E_2_	1.00	1.00–1.01	< 0.001	LH	1.14	1.08–1.20	< 0.001
T	0.60	0.42–0.87	0.007	T	1.54	1.06–2.24	0.025

*DOR* Diminished ovarian reserve. *AMH* Anti-Müllerian hormone. *PCOS* Polycystic ovarian syndrome. *BMI* Body mass index. *LH* Luteinizing hormone. *FSH* Follicle-stimulating hormone. *E*_*2*_ Estradiol. *T* Testosterone

Binary logistic regression with forward method was used to analyze the prediction on PCOS by age, weight, BMI, AMH, FSH, LH and T. Age, BMI, AMH, LH, and T, but not weight and FSH, were the significant factors which independently predicted the likelihood of PCOS. In a logistic regression model adjusted for age, BMI, AMH and LH, one nmol/L increase of T level was associated with 1.54 times increased (95% CI: 1.06–2.24, *P* = 0.025) risk of PCOS (Table 3).

## Discussion

In this study we demonstrated that age was moderately negatively correlated with AMH concentration. Risk of DOR increased and risk of PCOS decreased significantly with age. Since it is widely accepted that oocyte number and quality decline with age [16, 18], it is reasonable that age is a dominant factor of DOR. However, PCOS is considered a lifelong disease. What is the underlying reason for the decreased risks in aged population? In PCOS patients, due to the increased number of antral follicles, there is higher AMH concentration and lower basal FSH concentration compared to women with normal cycle [19], which leads to rare or no ovulation in PCOS. Aging leads to decreased follicle number, with decreased AMH concentration and increased basal FSH level, which is in favor of follicular growth and regular ovulation [20]. Therefore, it is possible that the diagnosis of PCOS cannot be made any more if it is not diagnosed in earlier age, so the incidence of PCOS decreased as age increased. In younger women with PCOS the main manifestations are hyperandrogenism and chronic anovulation, whereas in older women with PCOS, the main manifestations are obesity, insulin resistance, and metabolic disturbances [21]. A study comparing postmenopausal women with and without PCOS found that PCOS women had similar menopausal age, body weight, BMI, LH, total testosterone, and estradiol, except lower FSH [22]. In a word, the decreased risk of PCOS in aged population may be due to the difficulty of diagnosis.

In the present study, basal FSH was mildly correlated with AMH negatively. This result is in line with the previous studies. It is reported that there is a negative correlation between AMH concentration and serum FSH concentration in PCOS, and AMH may reduce the concentration of FSH and decrease follicle sensitivity to FSH by inhibition of aromatase [23, 24]. This study demonstrates that elevated FSH level is associated with increased risk of DOR, and this is generally accepted worldwide [16].

In this study basal LH was mildly correlated with AMH positively, and elevated LH level was associated with increased risk of PCOS. This result is consistent with the previous studies [25–27], indicating an interaction between AMH and LH secretion, which may contribute to the pathogenesis of PCOS [28]. On one hand, AMH activates the GnRH neuron through AMH receptors and increases GnRH-dependent LH pulsatility and secretion [10]. On the other hand, LH may increase AMH production in granulosa cells [29]. Therefore, increased AMH and LH stimulate each other mutually and play a role together in follicular arrest and pathogenesis of PCOS.

We found that T was mildly correlated with AMH positively. Moreover, elevated T level was associated with increased risk of PCOS and decreased risk of DOR. The positive correlation between serum AMH and total testosterone has been reported previously [24–26, 30]. It is demonstrated that androgens stimulate follicular FSHR expression, amplify FSH effect, promote follicular growth, which may lead to increased AMH production [31, 32]. Meanwhile, elevated AMH increases LH secretion in GnRH-neurone through AMH receptors [10], and elevated AMH inhibites aromatase expression in granulosa cells disturbing the transformation of androgen to estrogen [13, 33], which leads to the elevated androgen level. The two hormones may mutually reinforce each other, which may be the reason for the positive correlation of them. Elevated T level is associated with increased risk of PCOS. In PCOS with high androgen, serum AMH levels increase significantly compared with PCOS with normal androgen [27]. Others suggest that serum AMH level is related to the severity of PCOS [34, 35]. Such an interaction may contribute to the pathogenesis of PCOS [28].

**Fig. 4:**
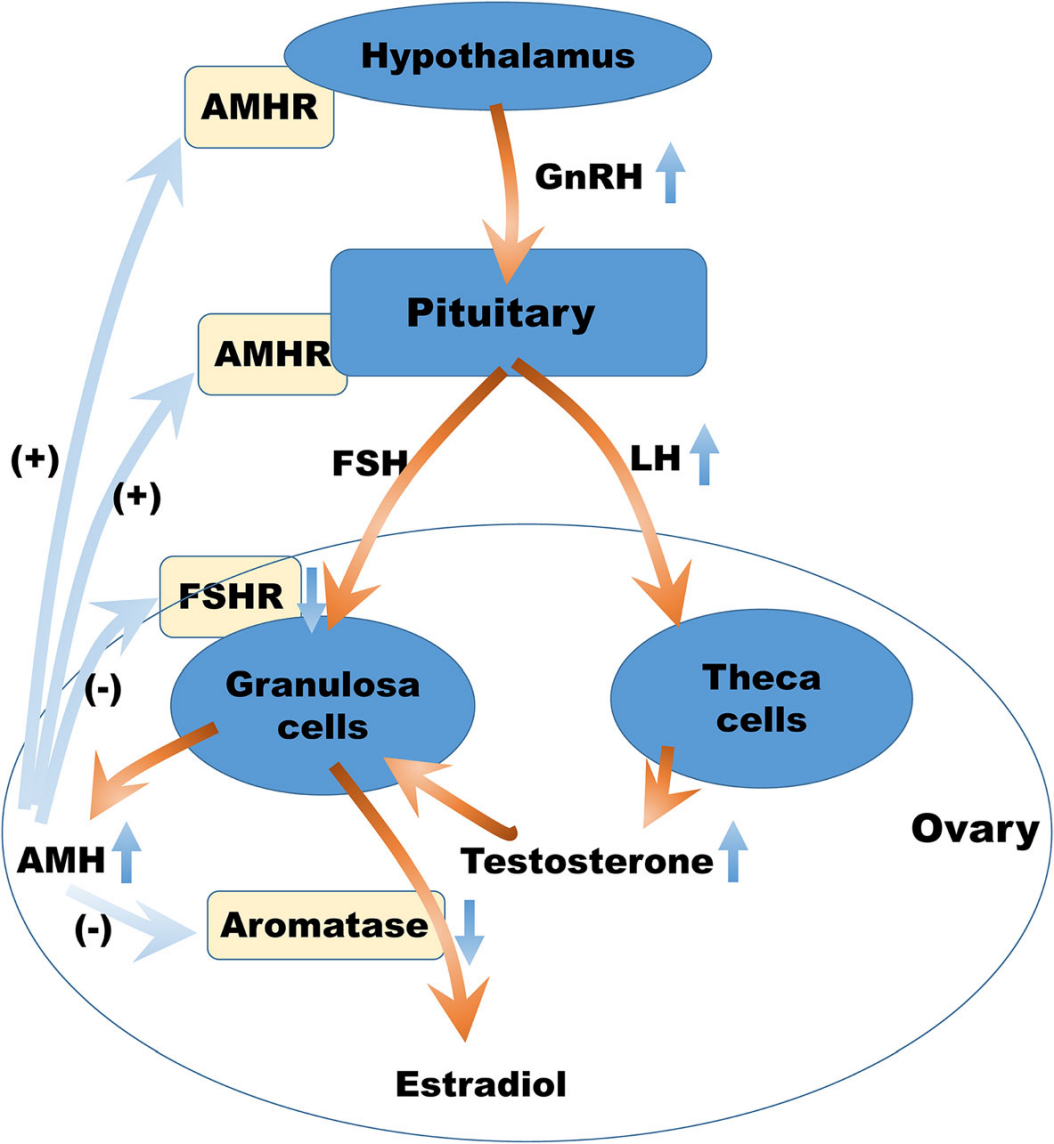
Schematic diagram of AMH and androgen in DOR and PCOS. In PCOS, elevated AMH stimulates LH expression by AMHR on hypothalamus and pituitary, increasing androgen secretion of theca cells. Meanwhile, elevated AMH inhibits FSHR expression and aromatase expression, which prevents the conversion of androgen to estrogen and follicle growth, leading to increase of androgen level. In return, elevated androgen stimulates AMH secretion of granulosa cells directly and indirectly. Therefore, elevated AMH and androgen promote mutually in a circle. In DOR, decreased AMH removes AMH induced inhibition of FSH expression, leading to faster conversion of androgen to estrogen and accelerated follicle consumption, which results in insufficient androgen and decreased ovarian reserve. In return, decreased androgen leads to decreased estradiol, elevated FSH, accelerated follicle consumption, and decreased AMH. AMH: anti-Müllerian hormone. DOR: diminished ovarian reserve. PCOS: polycystic ovarian syndrome. AMHR: receptor of AMH. FSH: follicle-stimulating hormone. LH: luteinizing hormone

As shown in Fig. 4, in PCOS high androgen leads to increased AMH level, and high AMH inhibits follicular development, leading to accumulation of small follicles without development of dominant follicles. High AMH decreases expression of FSHR and inhibit the response to exogenous gonadotropin, which leads to the poor response of ovarian stimulation. On one hand AMH impedes the conversion of androgen to estrogen, and on the other hand accumulated androgen leads to the increase of AMH, which thus initiates a vicious cycle. In DOR women, reduced androgen concentration accompanies with decreased AMH level. Insufficient androgen level cannot supply sufficient substrates for ovarian steroids synthesis. Meanwhile, decreased AMH level leads to accelerated follicular consumption and reduced ovarian reserve.

Either excess or deficiency of androgen may do harm to follicle development and deteriorate fertility [36]. In PCOS, it is demonstrated that suppression of androgen can improve the results of ovulation induction in PCOS [37, 38]. In DOR, androgen supplement may lead to a PCOS-like phenotype that increases oocyte production. Some randomized controlled trials (RCTs) have shown that DHEA and testosterone supplement can improve fertility in women with DOR [39, 40]. It is estimated that about a quarter of women with DOR are treated with DHEA supplements in all IVF centers [41]. Both literatures and our study point out that appropriate androgen level is required for normal follicular growth and is important in controlled ovarian hyperstimulation (COH) process.

The present study has some limitations. Firstly, in this study the diagnosis of DOR was made based on solely AMH. Until now, there is no uniformly accepted definition of DOR, as stated in the committee opinion provided by American Society for Reproductive Medicine [16]. There is mounting evidence to support the use of AMH as a screening test for poor ovarian response, but the data are insufficient yet [16]. In the Bologna criteria abnormal ovarian reserve test included AFC less than 5–7 follicles or AMH below 0.5–1.1 ng/ml [42]. The definition of DOR might be more solid with more criteria such as AFC. Secondly, since this is a retrospective observational study, we cannot distinguish other factors participating in follicular growth. Further RCTs and fundamental studies are required to confirm the roles of T in follicular growth and explore the underlying mechanisms. Furthermore, in infertile population with IVF we can develop an algorithm using AMH and T to predict ovarian response, which can help in daily work.

## Conclusions

In the present study, we found that age and FSH were main negative correlation factors, and LH and T were main positive factors of AMH. In DOR group, age, FSH and E_2_ increased and T decreased, while in PCOS group, LH and T increased and FSH decreased. Binary logistic regression found that age, weight, FSH, E_2_, and T were the significant factors which independently predicted the likelihood of DOR, and that age, BMI, AMH, LH, and T predicted the likelihood of PCOS. Since DOR and PCOS are manifested with insufficient and excessive AMH respectively, our study demonstrated that age and T were factors that most closely correlated with AMH level, and involved in DOR and PCOS. It is suggested that total testosterone plays an important role in follicular growth and more attention should be given to total testosterone level during COH process.

## Data Availability

The datasets used and/or analyzed during the current study are available from the corresponding author on reasonable request.

## Abbreviations

AMH: Anti-Müllerian hormone
AMHR2: AMH receptor type 2
BMI: Body mass index
COH: Controlled ovarian hyperstimulation
DOR: Diminished ovarian reserve
E_2_: Estradiol
FPG: Fasting plasma glucose
FPI: Fasting plasma insulin
FSH: Follicle-stimulating hormone
GnRHa: Gonadotropin-releasing hormone agonist
LH: Luteinizing hormone
P: Progesterone
PCOS: Polycystic ovarian syndrome
PRL: Prolactin
T: Testosterone
TGF-β: Transforming growth factor-β
